# Adherence to the Mediterranean diet partially mediates socioeconomic differences in leukocyte LINE-1 methylation: evidence from a cross-sectional study in Italian women

**DOI:** 10.1038/s41598-020-71352-9

**Published:** 2020-09-01

**Authors:** Andrea Maugeri, Martina Barchitta, Roberta Magnano San Lio, Giuliana Favara, Maria Clara La Rosa, Claudia La Mastra, Guido Basile, Antonella Agodi

**Affiliations:** 1 Department of Medical and Surgical Sciences and Advanced Technologies “GF Ingrassia”, University of Catania, Via S. Sofia 87, 95123 Catania, Italy; 2 Department of General Surgery and Medical-Surgical Specialties, University of Catania, Via S. Sofia 78, 95123 Catania, Italy

**Keywords:** Socioeconomic scenarios, DNA methylation, Risk factors, Epidemiology, Disease prevention, Nutrition, Public health

## Abstract

Although previous research demonstrated that socioeconomic status (SES) might affect DNA methylation, social inequalities alone do not completely explain this relationship. We conducted a cross-sectional study on 349 women (Catania, Italy) to investigate whether behaviors might mediate the association between SES and long interspersed nuclear elements (LINE-1) methylation, a surrogate marker of global DNA methylation. Educational level, used as an indicator of SES, and data on behaviors (i.e. diet, smoking habits, physical activity, and weight status) were collected using structured questionnaires. Adherence to Mediterranean diet (MD) was assessed by the Mediterranean Diet Score (MDS). Leukocyte LINE-1 methylation was assessed by pyrosequencing. Mediation analysis was conducted using the procedure described by Preacher and Hayes. Women with high educational level exhibited higher MDS (β = 0.669; 95%CI 0.173–1.165; *p* < 0.01) and LINE-1 methylation level (β = 0.033; 95%CI 0.022–0.043; *p* < 0.001) than their less educated counterpart. In line with this, mediation analysis demonstrated a significant indirect effect of high educational level on LINE-1 methylation through the adherence to MD (β = 0.003; 95%CI 0.001–0.006). Specifically, the mediator could account for 9.5% of the total effect. To our knowledge, this is the first study demonstrating the mediating effect of diet in the relationship between SES and DNA methylation. Although these findings should be confirmed by prospective research, they add value to the promotion of healthy dietary habits in social disadvantaged people.

## Introduction

Social determinants can explain health inequalities between and within countries, which should be tackled through Public Health interventions^1^. In general, low socioeconomic status (SES)—expressed in terms of educational level, employment and income—is associated with earlier onset of age-related chronic diseases and higher risk of death^2,3^. Socio-economic disadvantaged individuals tend to fare worse with regards to non-communicable diseases (NCD) risk factors, which include unhealthy behaviors^2–6^. Thus, strategies aiming to reduce health disparities should also include the promotion of healthy behaviors (e.g. diet, smoking habits and physical activity), which are considered as risk factors for several health conditions and diseases^7^.

Social disadvantages and unhealthy behaviors—occurring either in utero and during lifetime—may induce sustainable biological changes involved in individual NCD risk profile^8,9^. Despite recent strides in this field of research, molecular mechanisms involved are still not fully understood. For this reason, uncovering the epigenetic mechanisms underpinning this relationship might offer a plausible explanation of the effect of SES on human health. For instance, several lines of evidence have demonstrated that socioeconomic disadvantage significantly affected DNA methylation process, which resulted in aberrant gene expression and genome instability involved in chronic diseases and aging^10^. Social inequalities alone, however, do not completely explain socioeconomic difference in DNA methylation level, and further studies are required to unveil what factors contribute to it. Recent studies have also suggested that behaviors had different effects on global DNA methylation in exposed individuals and in future generations^11^. Some of these findings were obtained using the methylation status of long interspersed nuclear elements (LINE-1) sequences as a surrogate marker of global DNA methylation. Despite doubts about its validity as surrogate marker, aberrant methylation of these sequences might affect their independent and autonomous retro-transposition, leading to chromosomal instability and altered gene expression^12,13^. Moreover, previous evidence from preclinical and epidemiological studies suggested how dietary factors (i.e. nutrients, foods, and dietary patterns)^14–18^, smoking habits^19,20^, physical activity^21^ and weight status^22^ might affect DNA methylation process and LINE-1 methylation levels.

In this scenario, our hypothesis was that the behaviors (i.e. diet, physical activity, smoking habits and weight status) might mediate the association between SES and LINE-1 methylation. With this in mind, we conducted a cross-sectional study on women from Catania (Italy) to assess socioeconomic inequalities in LINE-1 methylation levels, and to examine whether behaviors were potential mediators of this difference.

## Results

### Study population

The current cross-sectional study included 349 women (aged 25–64 years), with a complete assessment of SES, behaviors and LINE-1 methylation. Overall, 25.5% of women were in the lower educational level, 47.6% were in the middle educational level and 26.9% were in the higher educational level. With respect to employment status, 54.2% of women were unemployed while 21.2% and 24.6% were part-time or full-time employed, respectively.

### Socioeconomic differences in LINE-1 methylation

**Figure 1:**
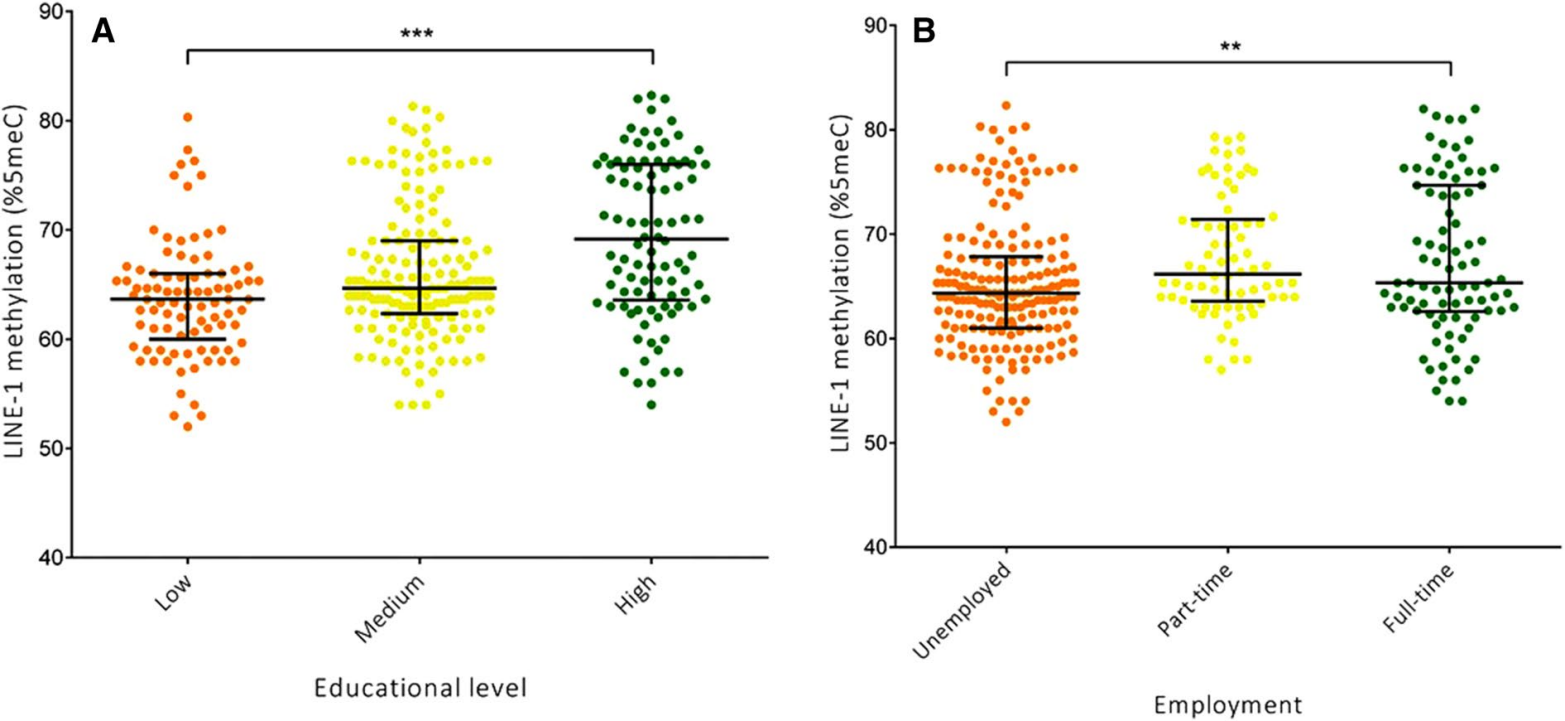
Comparison of LINE-1 methylation level by educational level (**A**) and employment status (**B**). **p < 0.01 and ***p < 0.001, based on the Kruskal–Wallis test.

We first compared LINE-1 methylation levels across categories of SES, expressed as educational level (Fig. 1a) and employment status (Fig. 1b). We observed that LINE-1 methylation level increased with increasing educational level (*p* < 0.001). Linear regression analysis on log-transformed data confirmed the increasing trend of LINE-1 methylation across educational levels in the age-adjusted model (β = 0.016; SE = 0.003; *p* < 0.001). Similarly, employed women exhibited higher LINE-1 methylation level than those who were unemployed (*p* = 0.002). This trend was confirmed by age-adjusted linear regression analysis on log-transformed LINE-1 methylation level (β = 0.007; SE = 0.002; *p* = 0.003). However, when SES indicators were evaluated simultaneously, only educational level maintained a statistically significant association with LINE-1 methylation (β = 0.016; SE = 0.003; *p* < 0.001).

### Association between educational level and behaviors

To evaluate whether behaviors mediate the effect of SES (using educational level as a proxy indicator) on LINE-1 methylation, we first compared BMI, MDS, smoking status and physical activity according to educational level (Table 1). Notably, BMI decreased with increasing educational level (*p* < 0.001), with highly educated women that were more likely to be normal weight (*p* < 0.001). Moreover, women with high educational level were less likely to perform physical activity (*p* = 0.012) and more likely to adhere to MD (*p* = 0.018) than their less educated counterpart. Instead, no social differences in smoking status were evident (*p* = 0.508).

**Table 1:** Population characteristics by educational level.

Characteristics^a^	Educational level			*p* value^b^
	Low (n = 89)	Medium (n = 166)	High (n = 94)	
Age, years	40 (27)	36 (25)	34 (17)	0.739
BMI	26.0 (7.5)	23.5 (4.9)	22.3 (5.0)	** < 0.001**
**BMI categories**				
Underweight	1.1%	7.9%	4.3%	** < 0.001**
Normal weight	42.0%	53.3%	69.9%	
Overweight	31.8%	28.5%	15.1%	
Obese	25.0%	10.3%	10.8%	
Mediterranean diet score	4 (3)	4 (3)	4 (3)	0.291
**MDS categories**				
Low	39.8%	42.7%	35.1%	**0.018**
Medium	52.4%	43.8%	50.0%	
High	7.8%	13.5%	14.9%	
**Smoking status**				
Current	20.5%	22.9%	15.1%	0.508
Former	15.9%	12.7%	11.8%	
Never	63.6%	64.5%	73.1%	
**Physical activity**				
Inactive	8.1%	19.4%	28.6%	**0.012**
Moderate active	91.9%	73.1%	68.6%	
Active	0%	7.5%	2.9%	

*p* values < 0.05 are indicated in bold font

^a^Results are reported as median (Interquartile range) or percentage.

^b^*p* values based on Kruskal–Wallis test for quantitative variables or Chi-squared test for categorical variables.

### The effects of behaviors on LINE-1 methylation

We next assessed the effects of behaviors on LINE-1 methylation using age-adjusted linear regression analyses. Interestingly, we observed a positive association between adherence to MD—expressed as MDS—and LINE-1 methylation (β = 0.006; SE = 0.001; *p* < 0.001). Moreover, former smokers (β = 0.014; SE = 0.007; *p* = 0.037) and non-smokers (β = 0.012; SE = 0.005; *p* = 0.020) exhibited higher LINE-1 methylation levels than current smokers. By contrast, no association with BMI and physical activity was evident (*p* values > 0.05). We also failed in demonstrating an interaction between adherence to MD and smoking status on LINE-1 methylation level (*p* value for interaction = 0.498).

### Mediation analysis

**Figure 2:**
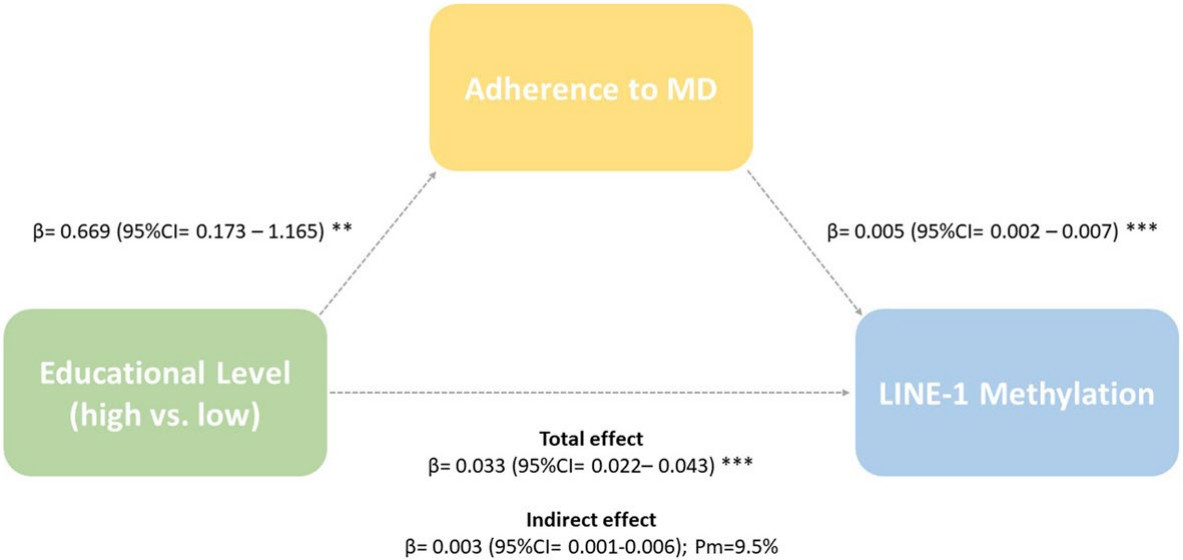
Analysis of the mediating effect of Mediterranean Diet Score in the association between high educational level and LINE-1 methylation. Mediation analysis was conducted using the procedure described by Preacher and Hayes^57^. Low educational level was used as reference group and age as covariate in all the regressions. Bias-corrected and accelerated bootstrap confidence intervals (CI) were calculated for indirect effects (a*b). Bootstrapping (5,000 samples) was conducted. The percentage mediated was expressed as the percentage of the total effect (path c) accounted for by the indirect effect (a*b). **p < 0.01 and ***p < 0.001.

Finally, we tested the mediating effect of behaviors on the relationship between educational level and LINE-1 methylation. Figure 2 shows positive associations of with high educational level with MDS (β = 0.669; 95%CI 0.173–1.165; *p* < 0.01) and LINE-1 methylation level (β = 0.033; 95%CI 0.022–0.043; *p* < 0.001). Notably, there was a significant indirect effect of high educational level on LINE-1 methylation through the adherence to MD (β = 0.003; 95%CI 0.001–0.006). The mediator could account for 9.5% of the total effect. Similar results were obtained using employment status as indicator of SES (Supplementary Fig. 1).

No mediation was evident when evaluating the indirect effect of medium educational level on LINE-1 methylation (β = 0.001; 95%CI − 0.001–0.004). A potential explanation is that medium educational level was not significantly associated with adherence to MD (β = 0.312; 95%CI − 0.127–0.752) (Supplementary Fig. 2). Similarly, none of the other behaviors were associated with a statistically significant mediation in the relationship between educational level and LINE-1 methylation (Supplementary Table 1).

## Discussion

The present study investigates potential behavioral mediators of socioeconomic differences in leukocyte LINE-1 methylation. Since LINE-1 elements are among the most common transposable sequences of the genome (with more than 500,000 copies), their methylation status has been widely used as a surrogate marker of global DNA methylation^23^. The maintenance of DNA methylation of CpG sites within LINE-1 sequences helps to prevent chromosomal instability, DNA rearrangement and alteration of gene expression^12,13^, which in turn might affect the risk for cancer, cardiovascular and neurodegenerative diseases^24–26^.

Among women without previous or current history of severe diseases, we first explored the association of SES and LINE-1 methylation, using educational level and employment status as proxy indicators. Notably, women in the highest socioeconomic classes exhibited higher LINE-1 methylation level than their disadvantaged counterpart. Yet, educational level seemed the strongest predictor of LINE-1 methylation when both SES indicators were evaluated simultaneously. This was in line with the current notion that life experiences—especially during the early life—affect epigenetic marks in specific loci of the genome^27^. In particular, several studies—mostly conducted on animals—showed that social stress, isolation, and contextual uncertainty were associated with changes in CpG methylation of promoter regions^27,28^. In addition, epidemiological studies demonstrated that adversity in early life was related to aberrant methylation profiles in adolescents and adults^29,30^. More recently, Fiorito and colleagues observed a graded relationship between SES—defined with various social indicators—and accelerated biological aging^31,32^. They obtained consistent results using two alternative predictors of accelerated biological aging based on DNA methylation at 353 CpG sites and 71 CpG sites, respectively^31,32^. These predictors, also known as ‘epigenetic clocks’, allow the estimation of biological aging as the difference between DNA methylation age and chronological age.

In our study, women with high educational level were also more likely to be normal weight, to perform more physical activity and to adhere more to MD than those with low educational level. For this reason, we also assessed whether lifestyle-related behaviors might affect LINE-1 methylation levels. In line with previous evidence^15,17,33^, we noted that women with high adherence to MD exhibited higher LINE-1 methylation levels that their unhealthy counterpart. These findings suggested that adherence to MD might partially explain and mediate socioeconomic differences in LINE-1 methylation level. To test this hypothesis, we assessed changes in the effect of educational level on LINE-1 methylation, adjusting for age (i.e. confounder) and adherence to MD (i.e. mediator). Specifically, adherence to MD was considered as a mediator since it was related to educational level and it was simultaneously associated with LINE-1 methylation. However, only ~ 10% of socioeconomic difference in LINE-1 methylation could be explained by adherence to MD, while residual difference was likely attributable to unmeasured factors. In fact, our analyses demonstrated that none of the other behaviors represented a potential mediator of the relationship between educational level and LINE-1 methylation. Similarly, the above mentioned study by Fiorito and colleagues aimed to investigate the role of behaviors in mediating the association between SES and accelerated biological aging^31,32^. However, their mediation analysis did not demonstrate a significant reduction of the association magnitude due to the inclusion of behaviors in the model^31,32^. They concluded that the association was robust to adjustment for mediators, with a partial effect attenuation when including smoking status in the model^31,32^. In our study, we observed that former and non-smokers exhibited higher LINE-1 methylation level than current smokers. Although mechanisms underpinning the effect of smoking on DNA methylation are not clearly understood yet, our findings were consistent with the belief that cigarette smoke is an environmental modifiers of DNA methylation^34^. However, cigarette smoking did non interact with adherence to MD, and therefore the latter cannot counteract the detrimental effect on LINE-1 methylation observed among current smokers. Moreover, the absence of relationship between educational level and smoking status ruled out one of the requirements for a significant mediating effect.

Our study had some limitations. Firstly, the cross-sectional design did not allow us to understand the causal link between diet and LINE-1 methylation. This is particular important when interpreting results from the mediation analysis. Indeed, mediation analysis has become a very popular approach and a potential alternative to causal inference methods^35^. Despite its promising perspectives, there are some concerns about its applicability to epidemiological data^36^. In our study, we evaluated all the preliminary requirements necessary to conduct a mediation analysis (i.e. evaluation of total effect and of all pathways involved in the mediating effect, comparison between direct and indirect effects, evaluation of alternative mediators)^35^. Yet in spite of this, our approach relied on cross-sectional data, which can lead to misrepresented conclusions if compared to longitudinal research^37^. From a biological point of view, the hypothesized pathway by which behaviors mediated the effect of social factors on DNA methylation was reasonable. However, caution is needed when interpreting our findings and further prospective studies on this topic should be encouraged.

Another limitation of our study regarded the debate on the potential application of LINE-1 methylation as a surrogate marker of global DNA methylation. In fact, heterogeneity in LINE-1 methylation levels across CpG sites and tissues^38–42^ hindered the comparisons between different studies. Despite these issues, aberrant LINE-1 methylation still remains an interesting molecular mechanisms in the research on cancer and other NCDs^24–26^.

Finally, data collection was performed by subjective methods that did not preclude errors and inaccuracy. In fact, the FFQ used for dietary assessment was prone to a degree of misreporting^43^. However, this tool has been developed and validated among women from Southern Italy^44^, and current findings were consistent with previous studies conducted on similar cohorts^45–49^. Moreover, we cannot evaluate the potential effect of additional social factors (e.g. income, household size and composition), confounders (e.g. genetic variants and environmental exposure), and mediators (e.g. drinking habits).

To our knowledge, this is the first study investigating the mediating effect of behaviors in the relationship between SES and LINE-1 methylation. Our findings confirm previous evidence that SES is a determinant of health, also through biological changes such as epigenetic mechanisms. Notably, we propose that behaviors—especially the adherence to MD—might mediate this association, leaving room for public health interventions aimed at promoting healthy dietary habits in social disadvantaged people. However, further prospective studies should be recommended to confirm this evidence taking into account additional social factors and behaviors.

## Methods

### Study design

The current cross-sectional study recruited women from those who referred for routine physical examination to three clinical laboratories in Catania (Italy) from 2010 to 2017. Pregnant women and those with an history of severe diseases, including cancer, diabetes, cardiovascular, neurodegenerative and autoimmune diseases, were excluded. Participants were fully informed of all aspects of the research protocol, which was conducted in accordance with the Declaration of Helsinki. All women were sufficiently literate to comprehend the research protocol and to sign a written informed consent to participate in the study. The protocol was approved by the ethics committees (Ethics Committees *“Catania”* and *“Catania 2”*) of the involved institutions (*Azienda Ospedaliero—Universitaria "Policlinico—Vittorio Emanuele"* and *Azienda Sanitaria Provinciale* of Catania, Italy) with the following protocol numbers: 52/2010/VE, 16/2015/CECT2, and 227/2011/BE. At recruitment, trained interviewers collected information on SES and behaviors using structured questionnaires. The current analysis included all the participants who provided a blood sample at recruitment. Each whole blood sample was centrifuged at 2,500 rpm for 15 min, and the buffy coat was immediately frozen at − 20 °C until further analyses.

### Socioeconomic status assessment

Women were asked to report their level of educational attainment and occupational position at recruitment. Educational level was categorized as low (primary school or none), medium (vocational or another secondary school) or high (university or vocational postsecondary school) level. Employment status was categorized as full-time employment, part-time employment, and unemployment (including housewives and retired). For statistical analysis, educational level and employment status were used as proxies for SES^31,32^, with low educational level and unemployment as reference groups.

### Behavioral data collection

Height and weight were measured at recruitment using standardized procedures, and body mass index (BMI) was obtained as the ratio between weight (kg) and squared height (m^2^)^50^. Information on smoking habits were collected using a questionnaire, and women were classified as never, former and current smokers.

Physical activity was assessed using the long form of the International Physical Activity Questionnaire (IPAQ-L)^51^. Women were categorized as inactive (no moderate or vigorous activity), moderately inactive, moderately active, and active (≥ 150 min/week moderate or ≥ 75 min/week vigorous or ≥ 150 min/week moderate + vigorous), according to the American Heart Association criteria^52^. Dietary data were obtained using a semi-quantitative Food Frequency Questionnaires (FFQ), from which estimated consumption of foods and beverages in g/day was calculated as previously described^5,15,17,33^. Adherence to Mediterranean diet was assessed using the 9-point index of Mediterranean Diet Score (MDS) and categorized as low (MDS range: 0–3), medium (MDS range: 4–6), or high (MDS range: 7–9)^53,54^.

### LINE-1 methylation analysis

DNA samples were extracted from buffy coats using the QIAamp DNA Mini Kit (Qiagen, Italy) according to the manufacturer’s protocol. Methylation analysis was performed on three CpG sites within the LINE-1 sequence (GenBank Accession No. X58075)^17,33^ to allow comparison with our previous studies in this field of research. Bisulphite conversion of 40 ng of each DNA sample was performed using the EpiTect Bisulfite Kit (Qiagen, Italy). PCR was conducted in a reaction volume of 25 μl, which contained 1.5 μl of bisulfite-converted DNA, 12.5 μl of PyroMark PCR Master Mix 2 × , 2.5 μl of Coral Load Concentrate 10 × , and 2 μl of the forward primer (5′-TTTTGAGTTAGGTGTGGGATATA-3′) and the reverse-biotinylated primer (5′-biotin-AAAATCAAAAAATTCCCTTTC-3′) (0.2 μM for each)^15,17,33,55^. Hot start PCR conditions were as follows: 1 cycle at 95 °C for 15 min, 40 cycles at 94 °C for 30 s, 50 °C for 30 s, and 72 °C for 30 s, and a final extension at 72 °C for 10 min. As described elsewhere^17,55,56^, pyrosequencing of PCR product was performed with 0.3 mM of the sequencing primer (5′-AGTTAGGTGTGGGATATAGT-3′) using the PyroMark Q24 instrument (Qiagen, Italy). All assays were conducted in triplicate including positive (100% methylated DNA) and negative (0% methylated DNA) controls, while failed assays were repeated. Intra-observer coefficient of variability between replicates was 2.2% (SD = 1.0%), as previously reported^56^. For each CpG site, methylation level was calculated as the ratio between methylated cytosines and the sum of methylated and unmethylated cytosines. LINE-1 methylation level was computed as the average methylation level of the three CpG sites.

### Statistical analysis

Statistical analysis was performed using the SPSS software (version 21.0, SPSS, Chicago, IL). We first compared LINE-1 methylation across categories of SES proxies (i.e. educational level and employment status). Due to its skewness (*p* < 0.001 based on the Kolmogorov–Smirnov test), LINE-1 methylation level was expressed as median and interquartile range (IQR) and compared using the Kruskal–Wallis test. The association of SES proxies with LINE-1 methylation was examined by age-adjusted linear regression models using log-transformed LINE-1 methylation level as the outcome. SES proxies were first added to the model one-by-one, and then included simultaneously. In all the analyses, the lower SES categories were used as the reference to assess associations of high SES with LINE-1 methylation.

Next, we compared behavioral characteristics across different educational levels. Categorical variables were compared using the Chi-square test, while continuous variables were compared using the Kruskal–Wallis test. The association of behaviors with LINE-1 methylation was examined by age-adjusted linear regression models using log-transformed LINE-1 methylation level as the outcome. The predictors that were initially considered separately were BMI (continuous), smoking status (ordinal categorical: current, former, never), physical activity (ordinal categorical: active, moderately active, moderately inactive, inactive), and MDS (ordinal categorical score from 0 to 9). We also tested for interaction between predictors that were significantly associated with log-transformed LINE-1 methylation level in the abovementioned age-adjusted linear regression models.

To evaluate whether the association of educational level with LINE-1 methylation was mediated by behaviors, we performed a mediation analysis using the procedure described by Preacher and Hayes^57^. The first equation (path a) regressed the mediator (MDS) on the independent variable (educational level). The second equation (path b) regressed the dependent variable (log-transformed LINE-1 methylation) on the mediator. The third equation (path c’) regressed the dependent variable on the independent variable, adjusting for the effect of the mediator. Low educational level was used as reference group and age as covariate in all the mediation models. Bias-corrected and accelerated bootstrap confidence intervals (CI) were calculated for indirect effects (a*b). Bootstrapping (5,000 samples) was conducted. The percentage mediated was expressed as the percentage of the total effect (path c) accounted for by the indirect effect (a*b).

## Supplementary information

Supplementary Information.

## Data Availability

The datasets analyzed during the current study are available from the corresponding author on reasonable request.
