# Supplementary material for: Adherence to the Mediterranean diet partially mediates socioeconomic differences in leukocyte LINE-1 methylation: evidence from a cross-sectional study in Italian women
Source: Sci Rep. 2020 Sep 1;10:14360. doi: 10.1038/s41598-020-71352-9 (PMC7463235; doi:10.1038/s41598-020-71352-9)
Supplement: Supplementary file 1 — Supplementary Information. [file 41598_2020_71352_MOESM1_ESM.docx]

**Adherence to the Mediterranean diet partially mediates** **socioeconomic differences in leukocyte LINE-1 methylation: evidence from a cross-sectional study in Italian women**

Andrea Maugeri^1^, Martina Barchitta^1^, Roberta Magnano San Lio^1^, Giuliana Favara^1^, Maria Clara La Rosa^1^, Claudia La Mastra^1^, Guido Basile^2^, Antonella Agodi^1,*^

^1^ Department of Medical and Surgical Sciences and Advanced Technologies “GF Ingrassia”, University of Catania, Catania, Italy, Via S. Sofia 87, 95123 Catania, Italy

^2^ Department of General Surgery and Medical-Surgical Specialties, University of Catania, via S. Sofia, 78, 95123 Catania, Italy

*Corresponding Author:

Antonella Agodi, Department of Medical and Surgical Sciences and Advanced Technologies “GF Ingrassia”, University of Catania, Catania, Italy, Via S. Sofia 87, 95123 Catania, Italy; email: agodia@unict.it

**Supplementary Figure 1.** Analysis of the mediating effect of Mediterranean Diet Score in the association between employment status and LINE-1 methylation.

**
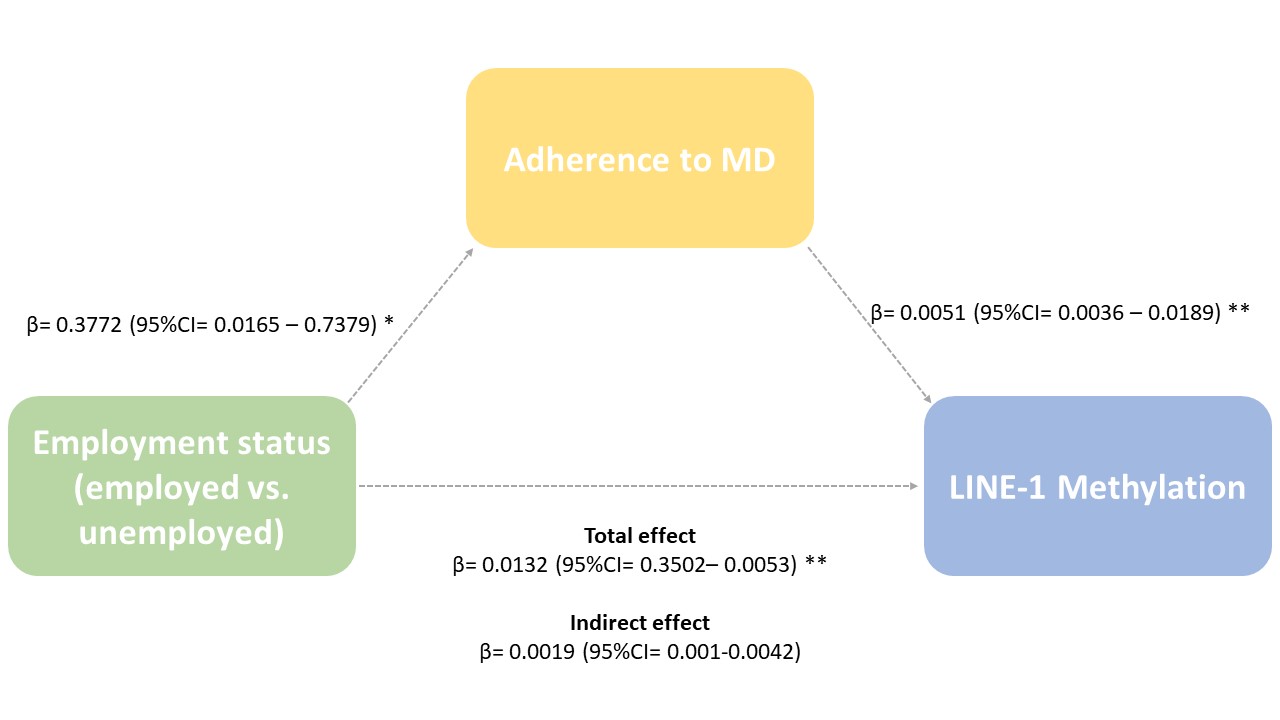
**

Mediation analysis was conducted using the procedure described by Preacher and Hayes. Unemployment was used as reference group and age as covariate in all the regressions. Bias-corrected and accelerated bootstrap confidence intervals (CI) were calculated for indirect effects (a*b). Bootstrapping (5000 samples) was conducted. * p < 0.05 and ** p < 0.01.

**Supplementary Figure 2.** Analysis of the mediating effect of Mediterranean Diet Score in the association between medium educational level and LINE-1 methylation.


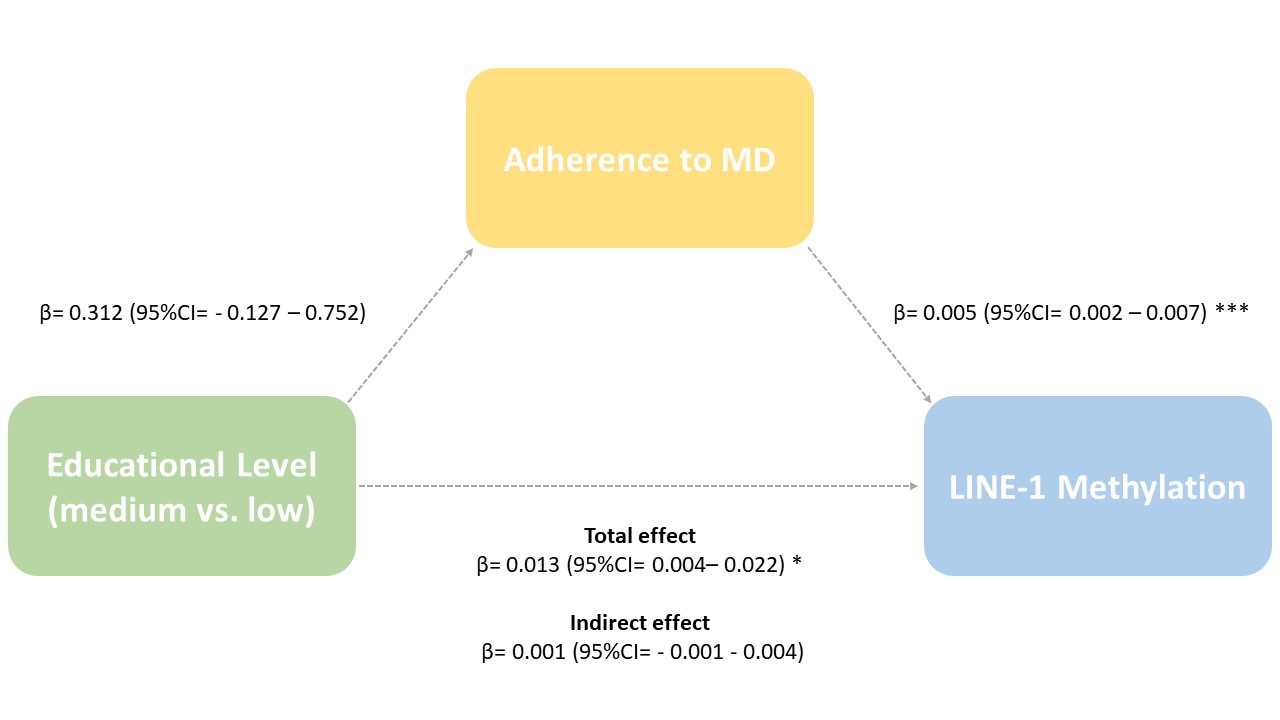


Mediation analysis was conducted using the procedure described by Preacher and Hayes. Low educational level was used as reference group and age as covariate in all the regressions. Bias-corrected and accelerated bootstrap confidence intervals (CI) were calculated for indirect effects (a*b). Bootstrapping (5000 samples) was conducted. * p < 0.05 and *** p < 0.001.

**Supplementary Table 1.** Analyses of the mediating effect of behaviors in the association between high educational level and LINE-1 methylation

| **Model^a^** | **Path a^b^** | **p-value** | **Path b^c^** | **p-value** | **Indirect effect^d^** |
| --- | --- | --- | --- | --- | --- |
| **BMI** | -3.507  (-5.351-1.663) | p<0.001 | 0.0002  (-0.0004-0.0008) | 0.513 | -0.0007  (-0.0027-0.0027) |
| **Smoking status** | 0.136  (-0.101-0.374) | 0.260 | 0.0044  (-0.0003-0.0090) | 0.065 | 0.0006  (-0.0005-0.0021) |
| **Physical activity** | -0.084  (-0.454-0.287) | 0.656 | -0.0023  (-0.0059-0.0013) | 0.229 | 0.0002  (-0.0009-0.0013) |

^a^ Models included educational level as dependent variable, each behavior as mediator, and log-transformed LINE-1 methylation level as dependent variable. Low educational level was used as reference group and age as covariate in all the mediation models.

^b^ Path a regressed the mediator on the independent variable. Results are reported as β coefficient and 95% confidence intervals.

^c^ Path b regressed the dependent variable on the mediator. Results are reported as β coefficient and 95% confidence intervals.

^d^ Bias-corrected and accelerated bootstrap confidence intervals (CIs) were calculated for indirect effect (a*b). Bootstrapping (5000 samples) was conducted.
